# Is home delivery really preferred? a mixed-methods national study in Pakistan

**DOI:** 10.1186/s12961-015-0039-2

**Published:** 2015-11-25

**Authors:** Shamsa Zafar, Siham Sikander, Ikhlaq Ahmad, Mansoor Ahmad, Nazia Parveen, Shumaila Saleem, Tayyba Nawaz, Zainab Suleman, Nadia Suleman, Noor ulAin, Ayesha Naeem, Asma Bashir

**Affiliations:** Human Development Research Foundation, Islamabad, Pakistan; Hno 9 B, Street 31, F8/1, Islamabad, Pakistan

**Keywords:** Birthing stations, Community midwives, Home delivery, Place of delivery

## Abstract

**Background:**

Pakistan has a high maternal mortality ratio and a low rate of skilled birth attendants (SBAs). To address these two important issues, the Pakistan Maternal Newborn and Child Health (MNCH) programme launched the community midwives (CMW) initiative in 2007. CMWs are supposed to conduct deliveries at community level outside health facilities. The purpose of the current study is to document perceptions about CMWs and preferences for birthing place.

**Methods:**

A mixed-methods study was conducted covering four provinces. For the quantitative survey, households were selected through a multistage sampling technique from rural districts. In 1,450 rural households, preferences of respondents about CMW-conducted deliveries were recorded. Qualitative data were obtained through focus group discussions (FGDs) and in-depth interviews (IDIs) with women, community elders, CMWs, and MNCH programme personnel in the same areas where the quantitative study was carried out. In both studies, preferences and the reasons behind particular respondent preferences were recorded. Frequencies of responses were analysed for the quantitative study. Narration and quotes from various types of participants were used to present findings from FGDs and IDIs.

**Results:**

In the quantitative study, 42% of respondents expressed a preference for birthing stations, i.e. a place where CMWs conduct deliveries; 22% preferred home deliveries. Birthing stations were favoured because of the availability of space and equipment and the proximity to women’s homes. These findings were largely supported by the qualitative component, although a range of views about where a CMW should conduct deliveries were expressed.

**Conclusion:**

Insights into where CMWs might provide delivery services were obtained through this study. Birthing stations may be an option as a preferred location for delivery care and should be considered as part of Pakistan’s national CMW programme.

## Background

Globally, a high proportion of women die due to pregnancy-related complications every year, with 98% of these deaths occurring in developing countries [1]. Pakistan, with a maternal mortality ratio of 276 per 100,000 live births [2], is one of the six countries contributing to 30% of the world’s maternal mortality [3]. National figures show that 52% of deliveries are conducted by skilled birth attendants (SBAs) in Pakistan [2]. Traditional birth attendants (TBAs) or un-trained family relatives also conduct deliveries at home. The situation is similar to other countries in the region [4–9]. In Pakistan, high maternal and neonatal mortality is being addressed by promoting deliveries with SBAs. However, accessibility is a problem and factors such as cost, transport, privacy, the comforts of delivering at home, family influence, and cultural traditions are often cited as barriers to their utilization [10].

Various projects in Pakistan, such as Hala and SMART Projects, have tried to increase the rate of deliveries by SBAs [11,12]. The Maternal Newborn and Child Health (MNCH) programme aims to increase SBA deliveries by introducing professionally trained community midwives (CMWs) for antenatal, labour, and postnatal care. Studies have explored the challenges faced by CMWs, which include unsatisfactory practical training, lack of supervision, monitoring, and finances, poor communication and coordination by officials of the health system, referral system barriers, and winning the trust of community, especially when experienced TBAs are available [13,14]. The general approach used is that CMWs will conduct deliveries in women’s homes. However, the idea of a birthing station has been piloted by certain organizations like the Pakistan Initiative for Mothers and Newborns [15]. These birthing stations are placed in the homes of CMWs, equipped with delivery kit and supplies.

The present study was carried out in four provinces in Pakistan to explore whether communities prefer home deliveries if a SBA (i.e. CMW), was well within reach and had an established birthing station. Previous national studies have highlighted that women prefer to deliver at home even if a free and accessible facility is available [15]. Findings from this study will enable the MNCH programme to align their placement strategy of CMWs with the needs and preferences of communities. In so doing, a higher degree of acceptance of the CMW initiative at the community level may be achieved. Considering recent devolution in Pakistan, we hope that this study will be a catalyst for reviewing the current provincial strategies for deployment of CMWs.

## Methods

This was a mixed-methods study conducted in the four provinces of Pakistan from March 2011 to February 2012. The quantitative component was a cross-sectional survey eliciting respondents’ opinions on CMWs and preferences for birthing place. The qualitative component explored the same issues, using FGDs and IDIs conducted in the same communities where the quantitative study was carried out.

### Quantitative component

The sample size for the quantitative study was calculated on the basis of 66% prevalence of home-based deliveries in Pakistan (from the 2008 Demographic and Health Survey) [2], at 95% confidence level, with a relative precision of 5%. A resulting total sample of 1,450 respondents was needed for national representation.

Multi-stage sampling was used. In the first stage, a list of MNCH districts where CMWs were deployed or were to be deployed was used as the sampling frame. From this list, 10% of districts from Pakistan’s four provinces were selected by simple random sampling. Three districts from Punjab (Attock, Layyah, Jehlum) and two each from Balochistan (Ziyarat and Quetta), Sindh (Tando Allah Yar and Sanghar), and Khyber Pakhtoonkhwa (KPK; Mansehera and Sawabi) were selected, representing a proportionate number of districts from each province relative to the size of the individual provinces. The second stage was to select 10% of rural union councils (the smallest administrative unit) from each included district. From the list of villages within a union council, one village was selected randomly. The last stage was to select eligible households with married women of childbearing age (15–49 years).

After entering the village, the research team started with the first household on the right side and approached every third house to inquire whether an eligible couple lived there. If the couple was not available, they moved to the next house to continue the process. As decisions on childbirth are collective family decisions, the household’s consensual perception was elicited and the term ‘respondent’ used in this paper could indicate the response of any member of the household.

A structured questionnaire was used to capture demographic data and responses on preference for place of birth and reasons for the preference. Provincial and national frequencies and percentages were calculated using STATA version 9.

### Qualitative component

A total of 20 FGDs and eight IDIs were conducted. Participants for FGDs and IDIs were selected from the same community where the quantitative study was carried out. FGDs and IDIs were held so as to be equally distributed across the four provinces and participants purposively selected to represent the different groups. Eight FGDs with eligible women (married women of childbearing age) were conducted, four with community elders (e.g. religious leaders, counsellors, and women elders) and eight with CMWs. Between 7 and 12 people were present for each of the FGDs, amounting to a total of 196 respondents. In addition, eight IDIs were conducted with MNCH programme personnel.

Separate field guides for IDIs and FGDs of different respondents were prepared and translated in to the local language. Audio recording and field notes were taken. The data collected was cleaned and transcribed. A grounded theory approach was used as a basis and data analysed manually using the constant comparative method. Themes were generated constantly and reflective sessions were done every other day to discuss emerging findings and questions were added in the field guides to incorporate those themes.

For both qualitative and quantitative components, training was provided to male and female data collectors, although, if there were cultural sensitivities, female data collectors were used. Data collection was supervised by two project coordinators in the field. Ethical approval was obtained from the National Bioethics Committee Pakistan–Research Ethics Committee before the start of the study. Written informed consent was obtained from the respondents prior to the quantitative data collection and verbal consent for FGDs and IDIs. Data cleaning and data entry were done in the central office and all data was anonymized and saved in files accessible only to the principal investigators.

## Results

### Quantitative component

A total of 1,457 eligible households were included. The study had a good response rate of 99.5%. The sociodemographic features of our national sample (e.g. women’s education, husband’s education, employment status, and parity) were comparable to the most recent National Demographic and Health Survey 2012–2013 [16].

Nationally, 42% of respondents expressed a preference for deliveries with CMWs in birthing stations, with this as the majority preference across all provinces (37% in Sindh, 53% in Punjab, and 39% in Balochistan and KPK). Both home and birthing place were expressed preferences in 29% and home was the preference in 22% nationally. In Sindh, Punjab, and Balochistan, a second preference was having an option of both birthing stations and home.

Table 1 shows the stated reasons for choosing birthing stations. The foremost perception was that the birthing station would have complete facilities which are not available at home. Lack of private and available space at home and proximity of the birthing station were other important factors expressed. There were some variations across provinces, for example, privacy and space at home seemed more important in KPK while proximity did not appear to be a consideration in Sindh.

**Table 1:** **Reasons for choosing birthing station as a place of delivery**

**Reported reasons for choosing birthing station** ^**a**^	**Sindh n = 131**	**Punjab n = 196**	**Khyber Pakhtunkhwa n = 142**	**Balochistan n = 140**	**National total n = 609**
Complete facilities at birthing stations	82%	87%	100%	94%	90%
No amenities at home	27%	67%	94%	49%	61%
Lack of private and available space for delivery at home	6%	41%	89%	27%	42%
Birthing station better equipped for emergencies	6%	64%	89%	41%	52%
Proximity of the birthing station	4%	60%	88%	64%	56%
Other reasons	0	36%	1%	4%	13%

^a^More than one reason could be given for choosing a particular preference so percentages amount to more than 100 [17].

Responses from households which preferred home-based deliveries by the CMW are provided in Table 2. Lack of finance was the most common reason for preference expressed on home deliveries at the national level (74%). Many perceived that deliveries at the birthing station would involve extra financial costs for medicine and (user) fees. A lack of transportation, stigma associated with women going out of the home, and privacy were also cited.

**Table 2:** **Reasons for choosing home as the place of delivery**

**Reported reasons for choosing home based deliveries** ^**a**^	**Sindh n = 85**	**Punjab n = 26**	**Khyber Pakhtunkhwa n = 120**	**Balochistan n = 84**	**National total n = 315**
Lack of finances	69%	69%	75%	77%	74%
Non-availability of timely transportation	27%	69%	68%	41%	50%
Family custom	46%	46%	26%	57%	41%
Birthing station will have costly medicine and higher fee	28%	65%	80%	76%	64%
Stigma	1%	11%	38%	27%	23%
Privacy	2%	62%	68%	53%	46%
Family support and help available at home	1%	42%	76%	47%	48%
Other reasons	4%	23%	0	13%	6%

^a^More than one reason could be given for choosing a particular preference so percentages amount to more than 100 [17].

### Qualitative component

Generally, the notion of having CMWs based in the communities was welcomed by women and elders across all the provinces. There was an instant and a very strong sense of ownership expressed from women respondents about the CMW initiative:

“*We will welcome the CMWs into our community as they will come here to provide a much needed service for us.*” (Female respondent) [17].

#### Preferences for birthing stations

Women and elders expressed preference for birthing stations as a place of delivery:

“*If we have CMWs and birthing stations in our communities we would have our children born at the best possible place. We have seen a lot of mothers and children die at the hands of TBAs and it is time to change this.*” (Male community elder) [17].

The reasons reported included availability of more equipment to handle unforeseen issues at the birthing stations, appropriate temperature of the environment, and better confidence levels of CMWs while providing services at her own place. Across all provinces, the major reason for choosing birthing stations was the perception of having better facilities compared to homes:

“*At the birthing station, the CMW will have proper instruments like medicines, oxygen, emergency kit and injections.*” (Female respondent) [17].

Having an optimum temperature during delivery was considered important for the health of the mother and newborn:

“*Our houses get very hot in summers and very cold in winters… It is very important for the health of the mother and the child that the room temperature is just right. This will not be a problem at the birthing stations.*” (Female respondent) [17].

Respondents expressed concerns that households do not have a room to spare at the time of delivery. As birthing stations would be catering for the deliveries, it was perceived that they would not have this problem:

“*Many houses do not have a separate room to have deliveries and it gets very difficult. As a result the women then opt to get delivered at their parent’s homes and that too is not always possible.*” (Female respondent) [17].

Women and community elders linked CMWs level of confidence and esteem to her place of work. They were of the opinion that CMWs would feel most comfortable at their birthing stations rather than in other people’s homes:

“*Mental satisfaction of CMW is also important while conducting deliveries and she will feel better at her own place.*” (Female respondent) [17]

Privacy at the birthing station was another reason expressed by most of the women:

“*The birthing station is better because to deliver at home in the presence of male members and children looks odd and embarrassing. Sometimes the woman starts crying which does not look good and children make fun of it afterwards.*” (Female respondent) [17].

CMWs themselves expressed a desire to establish birthing stations in their own homes because they feel more comfortable, secure, and confident at their own place.

In interviews with the MNCH programme personnel, it was clarified that the initial strategy or policy of the MNCH programme was to have CMWs conduct deliveries in women’s homes which would gradually replace women’s use of TBAs. However, some had different views:

“*CMWs should have a birthing station within her own house; we would want her to conduct deliveries there. This will help the CMWs as it will be very convenient for her. We are thinking of establishing birthing stations for them.*” (MNCH programme staff member) [17].

#### Preferences for deliveries in women’s homes

Some women and elders expressed a preference for deliveries at home conducted by a CMW. One of the reasons reported was related to their mother-in-law’s preference. The male family members usually do not interfere in this decision:

“*Mothers in law decide where to deliver, men do not know anything about it and they have nothing to do with it.*” (Female respondent) [17].

Cultural reasons, family restriction, and privacy were cited commonly in Balochistan and KPK:

“*I am not allowed to go out of the home even if I am dying.*” (Female from KPK) [17].

“*Delivery is a private and sensitive matter, so why make it public. It is not our practice to have our sisters and daughters go out for such matters. Homes are the most suitable place to be delivered even by a CMW.*” (Female from Baluchistan) [17].

MNCH programme personnel were of the opinion that CMWs should be conducting deliveries at women’s homes:

“*CMWs should go to homes for conducting deliveries.*” (District coordinator) [17].

There was recognition that, in certain areas, households may not have a proper place for conducting deliveries. The following suggestion was made:

“*CMWs should have a mobile cabin made of wood or plastic so that they can adjust that into the courtyard of houses because many houses in Balochistan and Khyber Pakhtunkhwa do not have surplus rooms*.” (District coordinator) [17].

#### Flexible options

There were some views which indicated that options should be available regarding birth place. Women expressed that it would be good to have the flexibility of being serviced by the CMWs at either the birthing stations or at their home. They indicated that this flexibility would allow the CMW to visit their homes and conduct the delivery in case of an emergency or when the delivery takes place at night:

“*We would prefer a flexible option that if we cannot go to the birthing station, for some reason or the other, the CMW should be able to come to our homes to provide delivery services.*” (Female respondent) [17].

The elders did not strongly opt for having the flexibility of services as described above and preferred that CMWs establish their birthing stations in the community:

#### Challenges faced by CMWs

CMWs related some of the barriers and challenges they faced. Lack of awareness in the community about CMWs, delay in their deployment and certification, inadequate remuneration of their services, mobility within the communities, young age, marital status, and competition with TBAs were expressed. CMWs reported that they did not feel ready in terms of knowledge and skill-set to start serving the communities:

“*We did not get a chance to practically conduct deliveries with the supervision of doctors due to which we are hesitant in going out and conducting deliveries.*” (CMW) [17].

“*We CMWs should be given training on presentation and communication skills that can be beneficial when visiting households. This will help us in introducing ourselves and engaging with the families in a more confident manner.*” (CMW) [17]

## Discussion

The MNCH programme in Pakistan is aiming to reduce maternal mortality by increasing deliveries with SBAs through the CMW programme. This study provides an important insight into the wishes of the community regarding CMWs and where they can provide delivery care. There is ample evidence suggesting that a majority of women in Pakistan deliver at home [2] and it is assumed that this would be the preference of the household – our study questions this notion. The major finding of this study was the predominant view that women should undergo childbirth at the birthing stations of CMWs. It is also heartening to note that community welcomes the idea of a CMW in their locality.

Other studies have indicated that economic and physical accessibility are key factors that contribute to choice of the place of birth [18,19]; the qualitative and quantitative findings of our study confirm that this is true in Pakistan. The factors do not operate across only the home-health facility spectrum, but also within the community, i.e. birthing at home and in a community-level birthing station. Our findings have shown that there is an unmet need for available, affordable, accessible, and community-based trained midwifery services. Expectations of what this service will provide are high (for example, well-equipped birthing stations and optimal physical conditions).

Nevertheless, not everyone prefers delivery in birthing stations. Divergent views have been captured from four types of respondents. Although many women, community elders, and CMWs preferred birthing stations for conducting deliveries, MNCH programme personnel have indicated that deliveries by CMWs have to be home-based. This suggests a need to reconsider the original design of the CMW programme. For the CMW initiative to be successful, it will be necessary to create a receptivity to preferences of community members and respond to expressed needs. However, there are signs of change as MNCH personnel had expressed the idea that a birthing station may be worth exploring. Flexibility in making the CMW available for births in women’s homes as well as in a birthing station may also be relevant.

This study also allowed some differences between the four provinces of Pakistan to be highlighted. Considering recent devolution in Pakistan, we hope that this study will be a catalyst for reviewing the current provincial strategies for deployment of CMWs. CMWs are the key stakeholders who will carry this initiative forward, provided their skills and motivation are fostered. This can be done by valuing their opinion regarding placements and by addressing identified challenges. A high standard skill-set is required, with sound practical experience for CMWs to work and communicate effectively in the community and the necessary strategies incorporated within programme activities.

A number of limitations arose in the conduct of the present study. We did not include the option for delivery in a health facility, therefore no comparisons can be made with preferences for this option. Our study focused, however, on the CMW, who is not providing services in health facilities. Although CMWs were included in the study, few were functional, and therefore the respondents were unlikely to have experienced CMW services. Further, the quantitative study relied on a ‘household response’ and therefore does not represent the views of a specific type of person within the household. We opted for this approach because decisions on delivery are usually collective family decisions. We acknowledge that recording bias may have occurred as well as subjectivity in the data collectors’ marked response if the complete household did not come to consensus. However, perceptions and opinions of different types of respondents (women and elders) were captured in the qualitative component. A large amount of data was collected in this study and, by presenting both qualitative and quantitative components, we have had to present our data selectively.

## Conclusion

This study provides some insights into community preferences relating to CMWs, with important implications for the MNCH programme. Alignment of policy and programme design with the preferences of the community and of CMWs is recommended. Specifically, the approach for CMWs to conduct home deliveries should be re-examined and options for using birthing stations considered. A flexible mixed approach with CMWs functioning in both settings may be optimal to cater for the preferences of the majority. CMWs will require continued skill development and motivational support. Now it is for the MNCH programme to capitalize on the opportunities identified and address the challenges foreseen by the stakeholders.

## Abbreviations

CMW: Community midwife
FGD: Focused group discussion
IDI: In-depth interviews
KPK: Khyber Pakhtoonkhwa
MNCH: Maternal newborn and child health
SBA: Skilled birth attendant
TBA: Traditional birth attendant
